# Sandwich-Type Electrochemiluminescence Immunosensor Based on CDs@dSiO_2_ Nanoparticles as Nanoprobe and Co-Reactant

**DOI:** 10.3390/bios13010133

**Published:** 2023-01-13

**Authors:** A-Ling Chen, Xiao-Yan Wang, Qing Zhang, Ning Bao, Shou-Nian Ding

**Affiliations:** 1 Jiangsu Province Hi-Tech Key Laboratory for Bio-medical Research, School of Chemistry and Chemical Engineering, Southeast University, Nanjing 211189, China; 2 Key Laboratory of Consumer Product Quality Safety Inspection and Risk Assessment for State Market Regulation, Chinese Academy of Inspection and Quarantine, Beijing 100176, China; 3 School of Public Health, Nantong University, Nantong 226019, China

**Keywords:** carbon dots, Ru(bpy)_3_^2+^, electrochemiluminescence, immunosensor

## Abstract

In general, co-reactants are essential in highly efficient electrochemiluminescence (ECL) systems. Traditional co-reactants are usually toxic, so it is necessary to develop new environmentally friendly co-reactants. In this work, carbon dots (CDs) were assembled with dendritic silica nanospheres (CDs@dSiO_2_ NPs) to form a co-reactant of Ru(bpy)_3_^2+^. Subsequently, a sandwich immunosensor for detecting human chorionic gonadotropin (HCG) was constructed based on CDs@dSiO_2_ NPs as co-reactants, the nanoprobe loaded with the secondary antibody, and Ru(bpy)_3_^2+^ as a luminophore. In addition, compared to directly as a signal probe, the luminophore Ru (bpy)_3_^2+^ as a part of the electrolyte solution is simpler in this work. The immunosensor has an extremely low limit of detection of 0.00019 mIU/mL. This work describes the synthesis of low-toxic, efficient, and environmentally friendly CDs, which have become ideal co-reactants of Ru(bpy)_3_^2+^, and proposes an ECL immunosensor with excellent stability and selectivity, which has great potential in clinical applications.

## 1. Introduction

Electrochemiluminescence (ECL) is a luminescence phenomenon resulting from electrochemical and chemiluminescence reactions on or near the electrode surface [1]. The ECL emitter produces redox products on the electrode surface when voltage is applied and then forms an excited state with some component of the system upon returning to the ground state emitting light radiation [2,3,4]. Because it does not require a light source, high sensitivity, and specificity, ECL technologies have distinct benefits over conventional optical techniques [5]. Due to the diversity of active materials and reactions, ECL has many applications, such as molecular identification, clinical diagnosis, and the detection of chemicals [4,6,7,8]. In general, the ECL process can be broadly categorized as an annihilation process and a co-reactant process [9,10]. The former has a large onset potential and a relatively modest ECL signal, and it is often operated in organic solvents [11]. In contrast to the annihilation process, the co-reactant process can overcome the narrow solvent potential window and the unstable radical ion and has a stronger ECL emission due to the introduction of a co-reactant. The co-reactant process is more widely used in the present ECL analytical field [12,13,14].

Ru(bpy)_3_^2+^ and its derivatives are currently the most popular studied ECL co-reactant system, because of their stable luminescence, low dosage, transparent luminescence mechanism, recoverability, and adaptability under different pH conditions [15,16,17,18]. There are many applications for Ru(bpy)_3_^2+^ and its derivatives, such as biosensors, immunoassays, food, and environmental detection [19,20]. In recent years, researchers have concentrated on the development of new co-reactants of Ru(bpy)_3_^2+^ and the application of this compound in analytical science. So far, it has been discovered that a wide variety of substances, including tri-n-propylamine (TPrA) [21], oxalic acid [22], methanol, amino acids, etc., can be employed as co-reactants of Ru(bpy)_3_^2+^. TPrA is the most widely used co-reactant in the Ru(bpy)_3_^2+^ system. However, the TPrA has some shortcomings, such as toxicity, corrosivity, and volatility, and the applicability of its sensors is limited [23]. Consequently, the quest for effective co-reactants of Ru(bpy)_3_^2+^ has become an important direction to broaden the application of ECL systems, and it also has significant theoretical and practical implications for the creation and improvement of ECL systems. There have been reports of using carbon dots (CDs) as co-reactants of Ru(bpy)_3_^2+^ in recent years. This nanomaterial benefits from good solubility, affordability, low toxicity, and strong chemical stability [24,25,26]. Carrara’s team found that amine-rich nitrogen-doped carbon nanodots (N-CDs) could be used as the ECL co-reactants of Ru(bpy)_3_^2+^ [27], and further constructed self-enhanced ECL systems by linking N-CDs with Ru(bpy)_3_^2+^ through covalent bonds to provide intramolecular electron transfer reactions. In another similar study, polyethyleneimine-encapsulated N-doped carbon dots (N-CDs@PEI) were proposed as co-reactants of Ru(bpy)_3_^2+^ nanosheets (Ru NSs) for ultrasensitive detection of DA [28]. However, the construction of an ECL sandwich immunosensor for human chorionic gonadotropin (HCG) detection using CDs not only as a co-reactant but also as a nanoprobe has not been reported.

**Figure 1:**
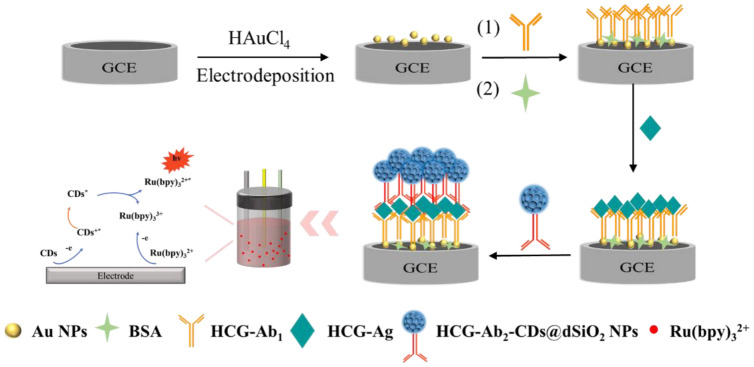
Schematic diagram of the preparation process and mechanism of the HCG immunosensor.

Here, an immunosensor for anodic ECL was developed for ultrasensitive diagnosis of HCG based on CDs-doped dendritic silica nanoparticles (CDs@dSiO_2_ NPs) as co-reactants of Ru(bpy)_3_^2+^. According to Figure 1, the first step was electrodeposition of gold nanoparticles (Au NPs) on glassy carbon electrodes (GCEs, 3 mm), and the HCG-Ab_1_ was immobilized on Au NPs by an Au-S covalent bond, and after specific capture of HCG, it was combined with HCG-Ab_2_ modified CDs@dSiO_2_ NPs (HCG-Ab_2_-CDs@dSiO_2_ NPs). In this ECL system, Ru(bpy)_3_^2+^ was uniformly dispersed in the buffer solution, and CDs@dSiO_2_ NPs were used as a nanoprobe as well as a novel co-reactant of Ru(bpy)_3_^2+^. The sandwich immunosensor demonstrated adequate selectivity, high sensitivity, and outstanding stability, which is expected to be used for the diagnosis of HCG in actual samples.

## 2. Materials and Methods

### 2.1. Materials

N-[3-(trimethoxysilyl) propyl] ethylenediamine (AEAPTMS), anhydrous citric acid, tetraethyl orthosilicate (TEOS), triethanolamine (TEA), N, N-dimethylformamide (DMF), tris(2-carboxyethyl) phosphine (TCEP), and Ru(bpy)_3_^2+^ were purchased from Sigma-Aldrich (St. Louis, MI, USA). Sodium salicylate, hexadecyl trimethyl ammonium bromide (CTAB), hydrochloric acid (HCl), aqueous ammonia (28%), chloroauric acid (HAuCl_4_∙4H_2_O), ethanol, sodium hydroxide, and succinic anhydride were supplied by Sinopharm Chemical Reagent Co., Ltd. N-Hydroxy-2,5-pyrrolidinedione (NHS) and (3-dimethylaminopropyl)ethyl-carbodiimidmonohydrochloride (EDC) were bought from Alfa Aesar Chemicals Co., Ltd. HCG antigen (Ag), HCG primary antibody (Ab_1_), and HCG secondary antibody (Ab_2_) were provided by Shanghai Jieyi Biotechnology Co., Ltd. (Shanghai, China). The phosphate-buffered saline solution (PBS, 0.1 M, pH = 7.4) was freshly prepared prior to use. Double-distilled water was used throughout.

### 2.2. Synthesis of Dendritic SiO_2_

The dSiO_2_ NPs were prepared through the use of the classical method [29]. Firstly, 68 mg of TEA was added to 25 mL of deionized water and stirred at 80 °C for 30 min. After that, 168 mg of sodium salicylate and 380 mg of CTAB were added and mixed for 1 h. Next, 4 mL of TEOS was quickly added to the mixture, and the reaction temperature was kept at 80 °C with a slight stirring for 2 h. After the reaction, 25 mL of ethanol was used to dilute the solution to terminate the reaction, and products were collected by centrifugation at 11,738× *g* for 5 min. To remove the residual organic template, the gathered precipitate was re-dispersed in a mixture of 6 mL of HCl and 30 mL of ethanol solution, and stirred at 60 °C for 12 h. After 5 min of centrifugation at 11,738× *g*, the precipitate was finally collected and washed repeatedly with ethanol and water. The obtained dSiO_2_ NPs were dispersed in 100 mL of ethanol for use.

### 2.3. Synthesis of CDs

A slightly improved method described in the literature was used to prepare CDs [30]. In brief, 15 mL of AEAPTMS was added into a 25 mL round-bottom flask under N_2_ flow, after the oxygen was removed completely, 1 g of anhydrous citric acid was added to the solution under vigorous stirring at 240 °C. The solution was continually heated for 5 min before being cooled to room temperature. The obtained products were centrifugated at 11,738× *g* for 5 min to remove large particles, and finally, the CD solution (63.2 μg/mL) was stored at 4 °C for further use.

### 2.4. Preparation of CDs@dSiO_2_ NPs

A 30 μL volume of the CD solution was added to 8 mL of the ethanol solution containing 10 mg of dSiO_2_ nanospheres and was then subjected to ultrasound for 5 min. This system was shaken at room temperature overnight. Then, the resulting CDs@dSiO_2_ NPs were dispersed in 5 mL of double-distilled water after four rounds of ethanol and deionized water rinsing.

### 2.5. Bioconjugation of CDs@dSiO_2_ NPs with Ab_2_

To obtain carbonylated CDs@dSiO_2_ NPs, 5 mg of CDs@dSiO_2_ NPs was dispersed in 5 mL of DMF, then the prepared dispersion was mixed with 25 mg of succinic anhydride and stirred for 2 h. Next, the solution was rinsed three times with deionized water and kept in 2.5 mL of water for further use.

The CDs@SiO_2_-Ab_2_ bioconjugates were prepared using our previous work [31]. In a nutshell, 500 μL of carbonylated CDs@dSiO_2_ NPs (2 mg/mL) was activated for 20 min with 15 μL of EDC (4.2 mg/mL), then shaken for 2 h at room temperature with 500 μL of Ab_2_ (10 μg/mL). The produced HCG-Ab_2_-CDs@dSiO_2_ NPs bioconjugates were repeatedly centrifuged and washed and then kept at 4°C in 1% BSA solution.

### 2.6. Fabrication of the ECL Immunosensor

Before use, the GCE was polished in turn with 0.3 μm and 0.05 μm alumina slurry, cleaned by ultrasound in ethanol and double-distilled water, then dried in N_2_ flow. Once the electrode had been cleaned, it was submerged in 1% HAuCl_4_ solution and electrodeposited at −0.25 V for 30 s to create an Au NPs substrate on the surface of the GCE. After 6 μL HCG-Ab_1_ was bound to the Au NPs by an Au-S covalent bond, it was incubated at 37°C for 100 min. Then, extra HCG-Ab_1_ was removed using the solution of PBS (0.01 M, pH = 7.4). To block non-specific adsorption sites, the electrode was then soaked in 1% BSA solution at 37°C for 1 h. Subsequently, PBS solution (0.01 M, pH = 7.4) containing different concentrations of HCG-Ag was dropped on the surface of modified electrodes and incubated for 80 min at 37°C. Finally, the HCG-Ab_2_-CDs@dSiO_2_ NPs were incubated on the formed electrode surface for 60 min, and the sandwich immunosensor was constructed. The ECL test of the immunosensor was performed in the solution of PBS (0.01 M, pH = 7.4) containing 50 μM Ru(bpy)_3_^2+^. The certificate of analysis of HCG-Ab_1_ and HCG-Ab_2_ is provided in Figure S1. Before use, 45 μL of TCEP solution (0.4 mM) was added to 45 μL of 0.2 mg/mL IgG antibody solution and incubated at 37 °C for 30 min to reduce the S-S bonds. All ECL tests were performed in a three-electrode system (Pt wire as the auxiliary electrode, saturated calomel electrode as the reference electrode, and GCE as the working electrode). The ECL tests were performed on an MPI-M ECL analyzer (Xi’an Remex Analytical Instrument Co., Ltd., Xi’an, China).

## 3. Results

### 3.1. Characterization of the CDs@dSiO_2_ NPs

**Figure 2:**
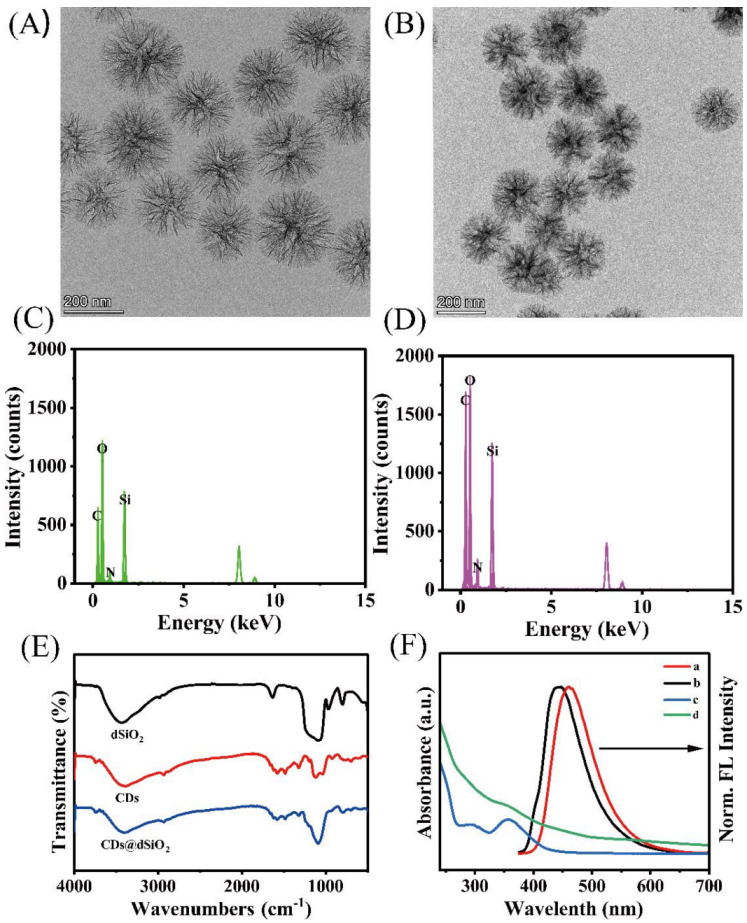
TEM images and EDX of (**A**, **C**) dSiO_2_, (**B**, **D**) CDs@dSiO_2_; (**E**) FTIR spectra of dSiO_2_ NPs, CDs, CDs@dSiO_2_ NPs, respectively, and (**F**) UV–vis absorption spectrum and fluorescence spectrum of (a, c) CDs, and (b, d) CDs@dSiO_2_ NPs.

The dSiO_2_ NPs and CDs@dSiO_2_ NPs were explored by TEM and EDX. As shown in Figure 2A, the synthesized dSiO_2_ NPs are monodisperse dendritic spheres with a well-defined “radial-porosity” central pore structure and an average diameter of 240 nm (Figure S2). The corresponding EDX mapping image (Figure 2C) shows a high content of Si and O elements. The dSiO_2_ NPs benefit from a large specific surface area, and the inner surface can be fully in contact with CDs. CDs were directly dispersed in the solution containing dSiO_2_ NPs to assemble the composite CDs@dSiO_2_ NPs. Figure 2B shows the TEM image of the composite CDs@dSiO_2_ NPs did not differ from dSiO_2_ NPs in appearance, which indicated that the growth of CDs did not destroy the structure of dSiO_2_ spheres and had a bare effect on the dispersion of the spheres. The EDX mapping image (Figure 2D) shows a significant increase in the content of the C element, indicating that the composite was successfully constructed. The Fourier transform infrared (FTIR) spectra (Figure 2E) were used to demonstrate the successful synthesis of CDs@dSiO_2_ NPs. For the dSiO_2_ NPs, in addition to the peak corresponding to the Si-OH stretching vibration at 960 cm^−1^, there are unique Si-O-Si stretching vibrations at 1080 cm^−1^ and 798 cm^−1^. For CDs, there is the stretching vibration peak of Si-O at 1033 cm^−1^ and 1119 cm^−1^. The broader peak at 3375 cm^−1^ represents the stretching vibration peak of O-H, indicating a large number of hydroxyl groups on the surface of the synthesized CDs, which makes the prepared CDs highly water-soluble. Finally, in the CDs@dSiO_2_ NPs spectrum, there are obvious O-H stretching vibration peaks and Si-O-Si stretching vibration peaks, and there is no Si-OH stretching vibration peak of dSiO_2_ spheres. This is due to the formation of Si-OH bonds between the hydroxyl groups on the surface of the silica and the silanol generated by the hydrolysis of the silane methoxy group in CDs, indicating the successful preparation of the composite. The crystal structure of the prepared dSiO_2_ NPs and CDs@dSiO_2_ NPs was further characterized by XRD (Figure S3). There is no difference in the peak shape of the two samples, and both have a large broad peak at 22°. The broad peak is an amorphous peak belonging to the dSiO_2_ NPs. This result indicates that the combination of dSiO_2_ NPs and CDs does not affect the crystal form of dSiO_2_ NPs. CDs showed a maximum fluorescence emission peak at 463 nm (Figure 2F), while CDs@dSiO_2_ NPs were blue-shifted to 443 nm, which may be due to the CDs being embedded in the inner surface of dSiO_2_ NPs. The change in the surface structure of the CDs causes a change in the luminescence behavior, which leads to the blue shift of the emission peak. UV–vis absorption spectra indicated that two typical absorption peaks of CDs at 291 nm and 356 nm belong to the π-π* leap of the C = C bond and the n-π* leap of the C = O bond, respectively. It can be seen from Figure S4 that dSiO_2_ NPs have almost no obvious absorption peak, indicating that the absorption in CDs@dSiO_2_ NPs mainly came from the successful loading of CDs. In the CDs@dSiO_2_ NPs, the CDs’ absorption peaks were almost unchanged. The change in the shape of the absorption spectra can be attributed to the scattering effect of the dSiO_2_ template. From Figure S5 can see that the CDs loaded into dSiO_2_ NPs, centrifuged, and the fluorescence intensity of the supernatant was lower than the original CD solution. It can be measured that 0.152 mg of CDs can be loaded on 1 mg of dSiO_2_ NPs. As shown in Figure S6, the storage under air conditions has little effect on the fluorescence emission of CDs@dSiO_2_ NPs, indicating the prepared CDs@dSiO_2_ NPs have good stability.

### 3.2. The Feasibility of CDs@dSiO_2_ NPs as Co-Reactants of Ru(bpy)_3_^2+^

**Figure 3:**
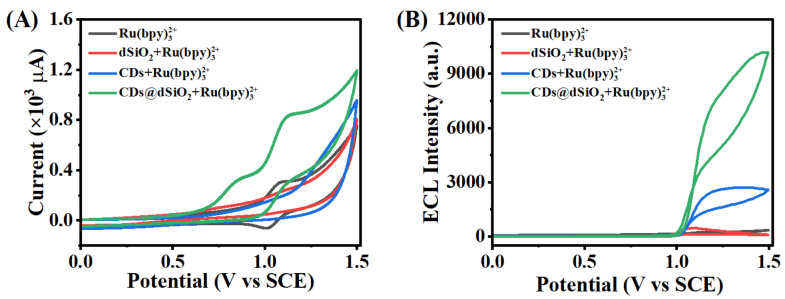
(**A**) CV curves and (**B**) ECL behaviors of Ru(bpy)_3_^2+^ (black curve), dSiO_2_-Ru(bpy)_3_^2+^ (red curve), CDs-Ru(bpy)_3_^2+^ (blue curve), and CDs@dSiO_2_-Ru(bpy)_3_^2+^ (green curve) in a solution of 0.1 M PBS (pH = 7.4) containing 50 μM Ru(bpy)_3_^2+^. The voltage of PMT was set at 600 V.

To investigate the feasibility of CDs@dSiO_2_ NPs acting as co-reactants in the classical Ru(bpy)_3_^2+^ system, ECL spectroscopy and cyclic voltammetry (CV) were carried out on CDs@dSiO_2_ NPs-modified GCEs. As shown in Figure 3A, Ru(bpy)_3_^2+^ solution (black curve) showed a reversible redox with a formal potential of + 1.07 V. The oxidation peak of Ru(bpy)_3_^2+^ rose and the reduction peak almost vanished when CDs@dSiO_2_ NPs were introduced to the system (blue curve). As a result, it was shown that oxidation of CDs was accelerated by electrogenerated Ru(bpy)_3_^3+^. Figure 3B depicts the ECL phenomenon of this system. It can be seen that the system containing only 50 μM Ru(bpy)_3_^2+^ in a buffer solution exhibits only a weak anodic ECL signal (black curve). Similarly, the dSiO_2_-Ru(bpy)_3_^2+^ system shows a weak signal (red curve). When CDs are introduced into the Ru(bpy)_3_^2+^ system, the signal is significantly enhanced, which indicates that CDs act as a co-reactant of Ru(bpy)_3_^2+^ (blue curve). Correspondingly, due to the enrichment of CDs by dSiO_2_ NPs, the ECL signal of the Ru(bpy)_3_^2+^-CDs@dSiO_2_ system are further enhanced. According to the above, the anodic ECL signal of the Ru(bpy)_3_^2+^-CDs@dSiO_2_ system was generated by Ru(bpy)_3_^2+^ and increased by the co-reactant CDs@dSiO_2_ NPs. These results support the notion that CDs serve as a co-reactant, similar to the typical co-reactant of TPrA. In addition, we also compare the differences in the properties of CDs@dSiO_2_ NPs and TPrA as co-reactants. From Figure S7 we can see that the ECL efficiency of CDs@dSiO_2_ NPs (15.62 mg/mL) was similar to the TPrA (2.03 mg/mL) in the solution 50 μM Ru(bpy)_3_^2+^. However, compared with TPrA, the CDs have good biocompatibility, high stability, and good water solubility [32].

Based on the above discussion, Ru(bpy)_3_^2+^ was oxidized to Ru(bpy)_3_^3+^, while CDs were oxidized to CDs^+*^, and a reductive intermediate CDs^*^ was created by the deprotonation procedure. The oxidized state Ru(bpy)_3_^3+^ reacted rapidly with the reduced state CDs* through high energy electron transfer to obtain the excited state Ru(bpy)_3_^2+^*. When electronic transition occurred, the excited state Ru(bpy)_3_^2+^* returned to the ground state so that an ECL signal can be observed. The specific ECL mechanism is described below:

Ru(bpy)_3_^2+^ − e- → Ru(bpy)_3_^3+^, (1)

CDs − e^-^ → CDs ^+^*, (2)

CDs ^+*^ − H^+^ →CDs *, (3)

CDs * + Ru(bpy)_3_^3+^ → Ru(bpy)_3_^2+^ + h, (4)

### 3.3. Construction of the ECL Biosensor

**Figure 4:**
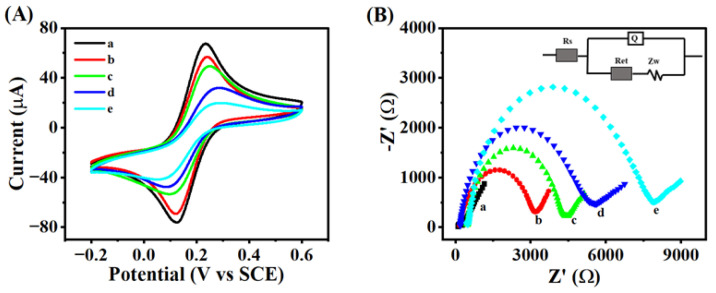
Electrochemical characterization of the preparation process of sandwich immunosensors: (**A**) CV and (**B**) EIS of (a) bare GCE, (b) Ab_1_/Au/GCE, (c) BSA/Ab_1_/Au/GCE, (d) Ag/BSA/Ab_1_/Au/GCE, and (e) Ab_2_-CDs@dSiO_2_ NPs/Ag/BSA/Ab_1_/Au/GCE (inset is the circuit model) measured in a solution containing 5 mM [Fe (CN)_6_]^3-^/^4-^ and 0.1 M KCl.

The Ru(bpy)_3_^2+^-CDs@dSiO_2_ system was used for extremely sensitive HCG detection because of its remarkable ECL performance. From the current–time curve in Figure S5, it can be obtained that the deposition charge of gold electrodeposition on the GCE surface is 0.006 C, and the theoretical deposition of Au NPs on the electrode surface was calculated to be 15.62 μg. Morphology of modified electrode surface studied by scanning electron microscope (SEM). The SEM images of bare GCEs and electrodeposited gold nanoparticles are shown in Figure S6. The results show a dense and uniform gold nanoparticle layer on the surface of GCEs, resulting in enhanced conductivity and increased surface area for capturing more HCG-Ab_1_. The absorbance of the UV–vis absorption spectrum of HCG-Ab_1_ and the solution containing the excess HCG-Ab_1_ of the constructed immunosensor was measured by using a spectrophotometer at 280 nm (Figure S7). It can be calculated that the amount of HCG-Ab_1_ immobilized on Au NPs was 3.58 μg. To investigate the modification process of biosensors, CV curves of various substances modified electrodes were collected. As displayed in Figure 4A, due to the weak electron transport capacity of Au-Ab_1_, the redox current decreased significantly when it was incubated on the surface of GCEs (Curve B). After Au-Ab_1_ was blocked by non-electroactive BSA, the conductivity of the modified electrode was decreased, and the current signal was further reduced (Curve C). Immediately after successive sandwich immune responses with HCG-Ag and HCG-Ab_2_-CDs@dSiO_2_ NPs, the peak current continued to decrease (Curves D and E), which was attributed to the formation of immune complex macromolecules with large spatial site resistance and weak conductivity. In addition, electrochemical impedance spectroscopy (EIS) was used to describe the process of electrode modification. The EIS was scanned at a direct current voltage potential of 0.18 V, an AC voltage amplitude of 0.005 V, an initial frequency of 100000 Hz, and a termination frequency of 0.01 Hz. As shown in Figure 4B, the continual change in the electron transfer resistance (Ret) on the surface of the electrode throughout the manufacturing process of the biosensor was consistent with the results of the CV. ECL signals were gathered for each preparation procedure in order to further examine the assembly process of the ECL immunosensor (Figure S8). As displayed in Figure S8 (Curves A–D), the ECL intensity is barely observed for bare GCEs, Ab_1_/Au/GCE, BSA/Ab_1_/Au/GCE, and Ag/BSA/Ab_1_/Au/GCE. Interestingly, the ECL signal significantly improved after the -Ab_2_-CDs@dSiO_2_ NPs/Ag/Ab_1_/Au/GCE was incubated on the electrode (Curve E). The Zeta potential was used to demonstrate the entire process of preparation of CDs@dSiO_2_ NPs and the combination with Ab_2_ (Figure S9). The surface of the produced dSiO_2_ NPs had a lot of hydroxyl groups, which resulted in a negative ζ potential. When a large number of CDs are embedded in dSiO_2_ NPs, the ζ potential of the composite becomes 15.5 mV. The surface charge becomes −45.7 mV after grafting the carboxyl group. Finally, the composite was incubated with positively charged Ab_2_, and the negatively charged CDs@dSiO_2_-COOH was partially neutralized. The results of Zeta potential further prove the synthesis of CDs@dSiO_2_ NPs and the smooth ligation of Ab_2_. These results prove that immunosensors were successfully constructed.

### 3.4. Optimization of Experimental Conditions

Factors affecting the biosensing process were optimized to ensure the optimal performance of the sandwich immunosensor. The pH of the PBS buffer solution has a noticeable impact on the ECL intensity. As displayed in Figure S10A, the ECL signal reaches its maximum at pH = 7.4, because either high acidity or high alkalinity will inactivate the protein and thus affect the antigen–antibody binding efficiency. Protein activity is significantly influenced by the temperature of the system. The temperature was set to 37 °C because it was more conducive to maintaining biological activity throughout the experiment (Figure S10B). In addition, the incubation time of Ab_1_ coupling with Au NPs and the specific binding time of Ag with Au-Ab_1_ are also important factors affecting the performance of biosensors. As can be seen from Figure S10C, ECL intensity was in a positive correlation with the incubation time of Au NPs and Ab_1_ until the plateau was reached after 100 min. Likewise, the incubation time of immunoreaction between Au-Ab_1_ and HCG-Ag was positively correlated with ECL intensity until it reached the maximum at 60 min and remained almost unchanged (Figure S10D). Therefore, in the following experiments, the incubation time of Au-Ab_1_ was selected as 100 min, and the time of Au-Ab_1_ coupling to HCG-Ag was selected as 60 min.

### 3.5. ECL Detection of HCG

**Figure 5:**
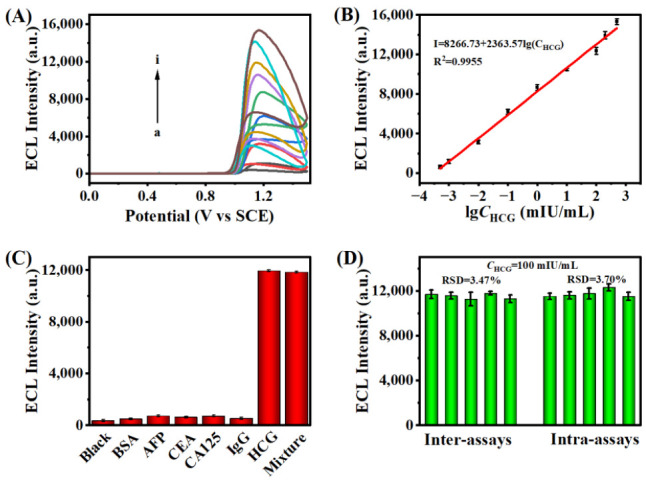
(**A**) ECL response with HCG of different concentrations (a to i: 0.0005 mIU/mL, 0.001 mIU/mL, 0.01 mIU/mL, 0.1 mIU/mL, 1 mIU/mL, 10 mIU/mL, 100 mIU/mL, 200 mIU/mL, 500 mIU/mL). (**B**) A linear relationship between the ECL intensities and lg*C*_HCG_. (**C**) Selectivity of the immunosensor with different interferences. (**D**) Reproducibility tests of the ECL immunosensor (intra-assays and inter-assays). (Error bars: SD, n = 3) in a solution of PBS (0.01 M, pH = 7.4) containing 50 μM Ru(bpy)_3_^2+^. The voltage of PMT was set at 800 V.

Under ideal conditions, various HCG concentrations were measured in order to assess the analytical performance of the ECL sandwich immunosensor. As shown in Figure 5A, the ECL signal gradually increased as the HCG concentration increased from 0.0005 mIU/mL to 500 mIU/mL. ECL intensity and HCG concentration had a very good linear relationship, as shown in Figure 5B. The linear regression equation was ECL intensity I = 8266.73 + 2363.57lgC_HCG_. The limit of detection of the immunosensor was 0.00019 mIU/mL (S/N = 3), and the R-squared correlation coefficient (R^2^) was 0.9955. The results indicate that the constructed sandwich immunosensor platform has satisfactory sensitivity. Additionally, compared to several existing detection approaches, the proposed ECL biosensor offered a wider linear range and a lower limit of detection (Table S1). These results indicate that the immunosensor has good application potential.

To demonstrate the selectivity of the immunosensor for HCG, common interferents such as alpha fetoprotein antigen (AFP), serum carbohydrate antigen 125 (CA125), carcinoembryonic antigen (CEA), and human immunoglobulin G (IgG) were tested as HCG surrogates and incubated at a concentration 10 folds higher than HCG (100 mIU/mL). As shown in Figure 5C, the sensing platform constructed with the interferents exhibited only weak ECL emission, which was not significantly different from the blank control. In contrast, a significant ECL response was detected for 100 mIU/mL HCG and for a mixture containing all interferents with 100 mIU/mL HCG, which demonstrated that only HCG can specifically bind to the vector. In the same condition, five glassy carbon electrodes were modified with the same concentration of HCG sensor to test ECL intensity (Figure 5D). The results reveale that there was barely any discernible difference in ECL signals between groups and within groups, indicating that the proposed biosensor had good reproducibility. As shown in Figure S11A, the ECL curves recorded by continuous 10 cycles scanning showed a low RSD value (1.5%), indicating that the immunosensor had reliable stability during the detection process. Storage stability is a key factor to evaluate the practicability of the constructed immunosensor. The GCEs with immunosensor modification were stored in a freezer at 4°C for one week, and the ECL intensity was recorded daily (Figure S11B), and the ECL signal showed only a slight decrease (RSD = 1.77%), which indicated the sensing platform had acceptable storage stability.

The feasibility of the proposed sandwich immunosensor for clinical application was assessed using a standard addition method. Healthy human serum was diluted 10,000 times with PBS (pH = 7.4, 0.1 mM). The ECL signals were tested by introducing different concentrations of antigen (5 mIU/mL, 15 mIU/mL, and 25 mIU/mL), and HCG concentration was calculated by a linear equation. As shown in Table S2, the recovery was 100–100.1%, and the RSD was less than 6.7%, which are satisfactory and show that the proposed ECL immunosensor is applicable for the detection of real samples.

## 4. Conclusions

To summarize, carbon dots proved to be excellent co-reactants and nanoprobes for Ru(bpy)_3_^2+^. The improvement of environment-friendly co-reactant makes the Ru(bpy)_3_^2+^-CDs@dSiO_2_ system have great potential in biosensing. In this sensing platform, dSiO_2_ NPs were used to load more CDs, and the prepared nanomaterials were used as the co-reactant of Ru(bpy)_3_^2+^ while acting as a nanoprobe to capture the HCG-Ab_2_, and Au NPs were used as a carrier to immobilize HCG-Ab_1_. This co-reactant was successfully applied to the ECL system of Ru(bpy)_3_^2+^ by sandwich immunoassay, and the whole immunoreaction process was carried out in an aqueous solution. An ultrasensitive detection of HCG was achieved with a linear range of 0.0005 mIU/mL to 500 mIU/mL and a calculated detection limit of 0.00019 mIU/mL. Specifically, it showed strong specificity, sufficient reproducibility and stability, and good detection recovery in real samples. Due to the good biocompatibility and water dispersibility of CDs, the Ru(bpy)_3_^2+^-CDs@dSiO_2_ ECL system is expected to be promising for biosensing.

## Data Availability

Not applicable.

## Supplementary Materials

The following supporting information [33,34,35,36,37] can be downloaded at: https://www.mdpi.com/article/10.3390/bios13010133/s1, Figure S1. The certificate of analysis of HCG-Ab_1_ and HCG-Ab_2_. Figure S2. TEM image of dSiO_2_ nanospheres at lower magnification and the corresponding particle size distribution (insert). Figure S3. Powder X-ray Diffraction Pattern of Powder X-ray Diffraction Pattern of dSiO_2_ NPs and CDs@dSiO_2_ NPs. Figure S4. UV–vis absorption spectra of dSiO_2_ NPs. Figure S5. Fluorescence spectrum of CDs and residual CDs in supernatant. Figure S6. Fluorescent stabilities of CDs@dSiO_2_ NPs against storage time. Figure S7. ECL curves of CDs and TPrA in the solution of PBS (0.1 M, pH = 7.4) containing 50 μM Ru(bpy)_3_^2+^. Figure S8. The current–time curve of electrodeposited Au NPs. Figure S9. SEM image of electrode surface GCE, Au NPs-GCE. Figure S10. UV–vis absorption spectrum of HCG-Ab_1_ and a solution containing the excess HCG-Ab_1_ of the constructed immunosensor. Figure S11. The ECL spectra of electrodes were modified with different substances. Figure S12. Zeta potential. Figure S13. Optimization. Figure S14. Performance stability and Storage stability of the ECL immunosensor. Table S1. Comparison of analytical performances with other reported HCG bioassays. Table S2. Analytical results for HCG detection in human serum specimens.
