# Supplementary material for: Sandwich-Type Electrochemiluminescence Immunosensor Based on CDs@dSiO2 Nanoparticles as Nanoprobe and Co-Reactant
Source: Biosensors (Basel). 2023 Jan 13;13(1):133. doi: 10.3390/bios13010133 (PMC9856027; doi:10.3390/bios13010133)
Supplement: Supplementary file 1 [file biosensors-13-00133-s001.zip › biosensors-2063058-supplementary.pdf]

Supporting Material

Sandwich-Type Electrochemiluminescence Immunosensor Based on CDs@dSiO2 Nanoparticles as Nanoprobe and Co-Reactant

A-Ling Chen [1], Xiao-Yan Wang [1], Qing Zhang [2], Ning Bao [3] and Shou-Nian Ding [1,*]

[1] Jiangsu Province Hi-Tech Key Laboratory for Bio-medical Research, School of Chemistry and Chemical Engineering, Southeast University, Nanjing 211189, China
[2] Key Laboratory of Consumer Product Quality Safety Inspection and Risk Assessment for State Market Regulation, Chinese Academy of Inspection and Quarantine, Beijing 100176, China
[3] School of Public Health, Nantong University, Nantong 226019, China
* Correspondence: snding@seu.edu.cn

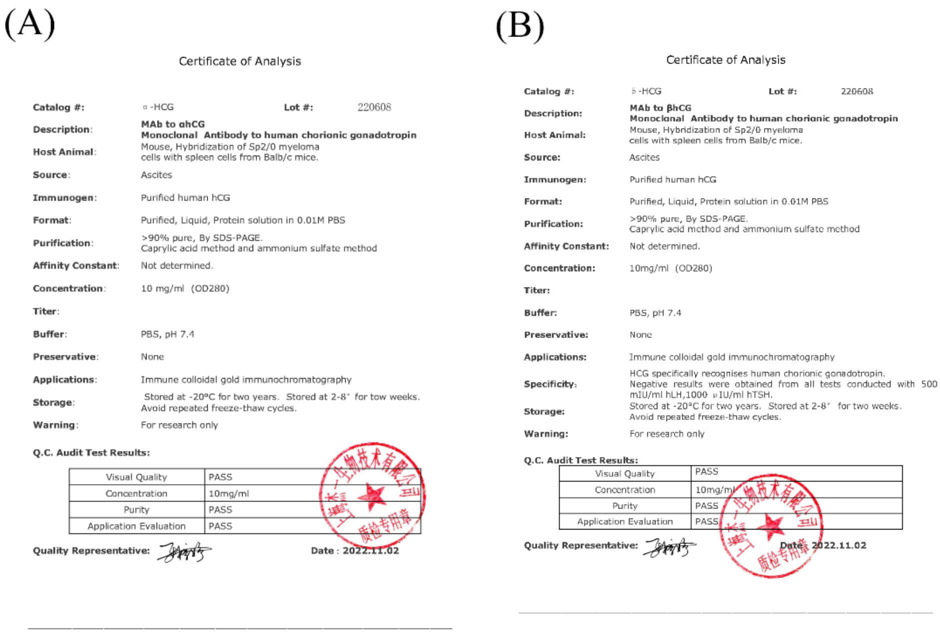

(A)

Certificate of Analysis

**Catalog #:**	α -HCG **Lot #:** 220608
**Description:**	**MAb to αhCG** **Monoclonal Antibody to human chorionic gonadotropin**
**Host Animal:**	Mouse, Hybridization of Sp2/0 myeloma cells with spleen cells from Balb/c mice.
**Source:**	Ascites
**Immunogen:**	Purified human hCG
**Format:**	Purified, Liquid, Protein solution in 0.01M PBS
**Purification:**	>90% pure, By SDS-PAGE. Caprylic acid method and ammonium sulfate method
**Affinity Constant:**	Not determined.
**Concentration:**	10 mg/ml (OD280)
**Titer:**	
**Buffer:**	PBS, pH 7.4
**Preservative:**	None
**Applications:**	Immune colloidal gold immunochromatography
**Storage:**	Stored at -20°C for two years. Stored at 2-8° for tow weeks. Avoid repeated freeze-thaw cycles.
**Warning:**	For research only

Q.C. Audit Test Results:

Visual Quality	PASS
Concentration	10mg/ml
Purity	PASS
Application Evaluation	PASS

Quality Representative: **Date : 2022.11.02**

(B)

Certificate of Analysis

**Catalog #:**	β -HCG **Lot #:** 220608
**Description:**	**MAb to βhCG** **Monoclonal Antibody to human chorionic gonadotropin**
**Host Animal:**	Mouse, Hybridization of Sp2/0 myeloma cells with spleen cells from Balb/c mice.
**Source:**	Ascites
**Immunogen:**	Purified human hCG
**Format:**	Purified, Liquid, Protein solution in 0.01M PBS
**Purification:**	>90% pure, By SDS-PAGE. Caprylic acid method and ammonium sulfate method
**Affinity Constant:**	Not determined.
**Concentration:**	10mg/ml (OD280)
**Titer:**	
**Buffer:**	PBS, pH 7.4
**Preservative:**	None
**Applications:**	Immune colloidal gold immunochromatography
**Specificity:**	HCG specifically recognises human chorionic gonadotropin. Negative results were obtained from all tests conducted with 500 mIU/ml hLH,1000 μIU/ml hTSH.
**Storage:**	Stored at -20°C for two years. Stored at 2-8° for two weeks. Avoid repeated freeze-thaw cycles.
**Warning:**	For research only

Q.C. Audit Test Results:

Visual Quality	PASS
Concentration	10mg/ml
Purity	PASS
Application Evaluation	PASS

Quality Representative: **Date: 2022.11.02**

Figure S1. The certificate of analysis of HCG-Ab1 and HCG-Ab2.

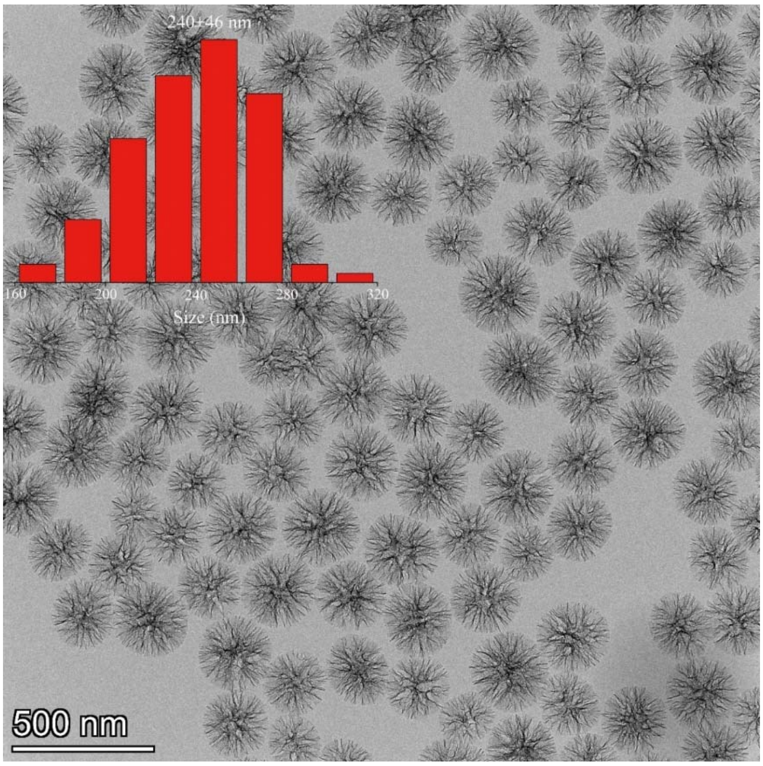

Figure S2. TEM image of $dSiO_2$ nanospheres at lower magnification. And the corresponding particle size distribution (insert).

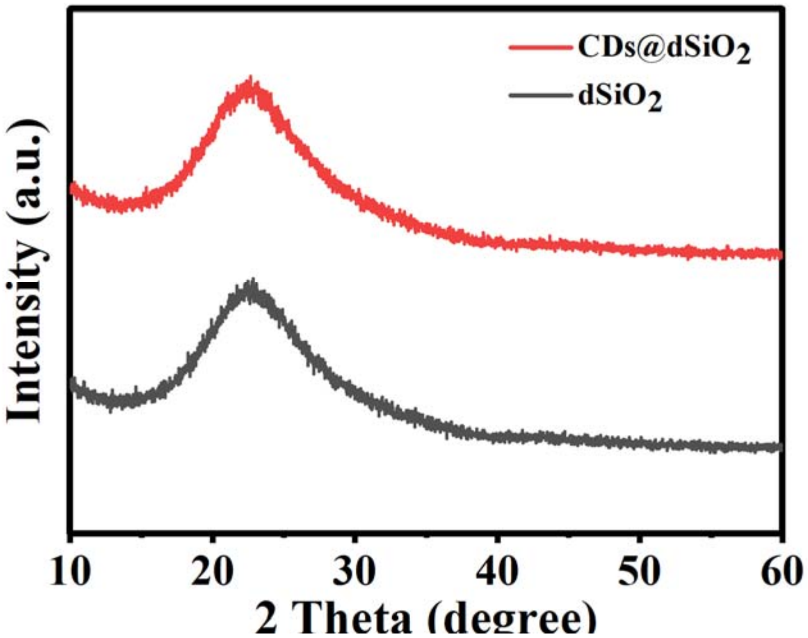

Figure S3. Powder X-ray Diffraction Pattern of $dSiO_2$ NPs (black cure) and CDs@$dSiO_2$ NPs (red curve).

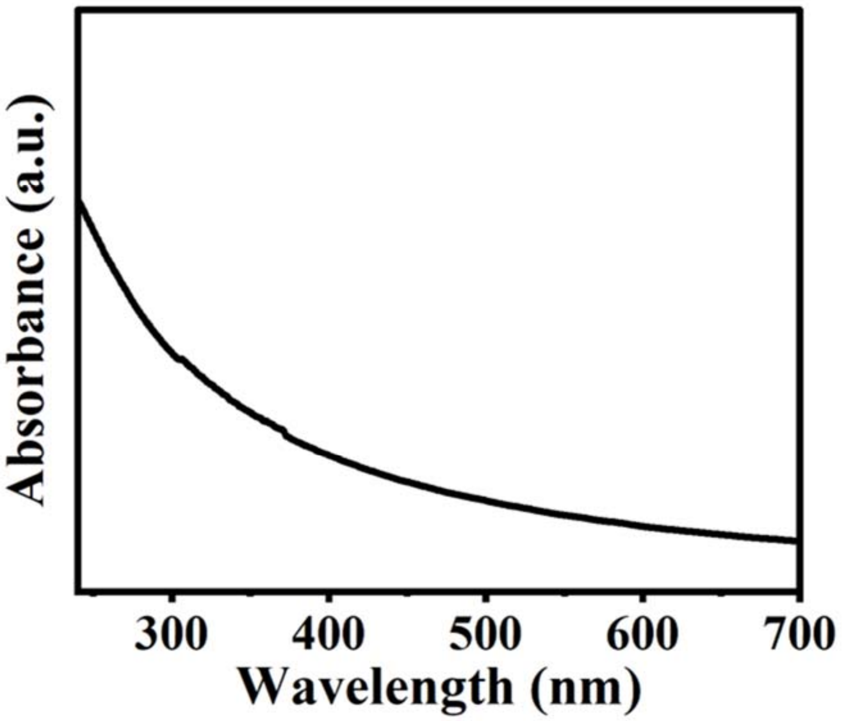

Figure S4. UV–vis absorption spectra of $dSiO_2$ NPs.

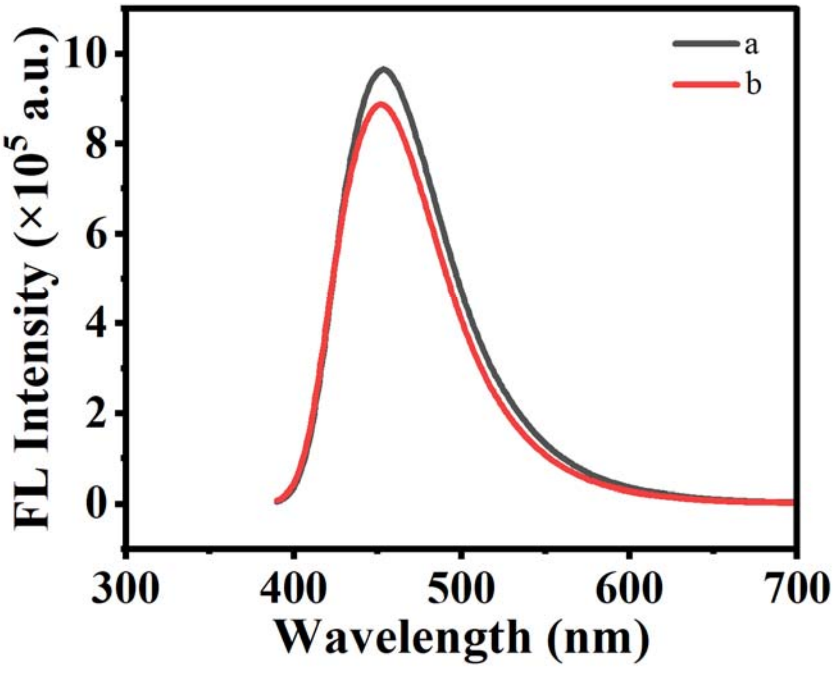

Figure S5. Fluorescence spectrum of (a) CDs and (b) residual CDs in supernatant.

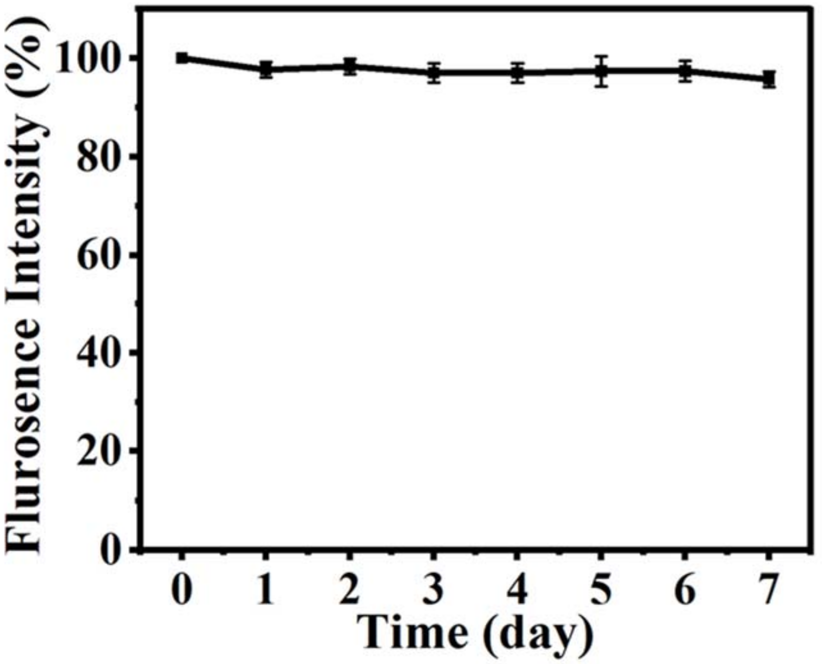

Figure S6. Fluorescent stabilities of CDs@dSiO$_2$ NPs against storage time.

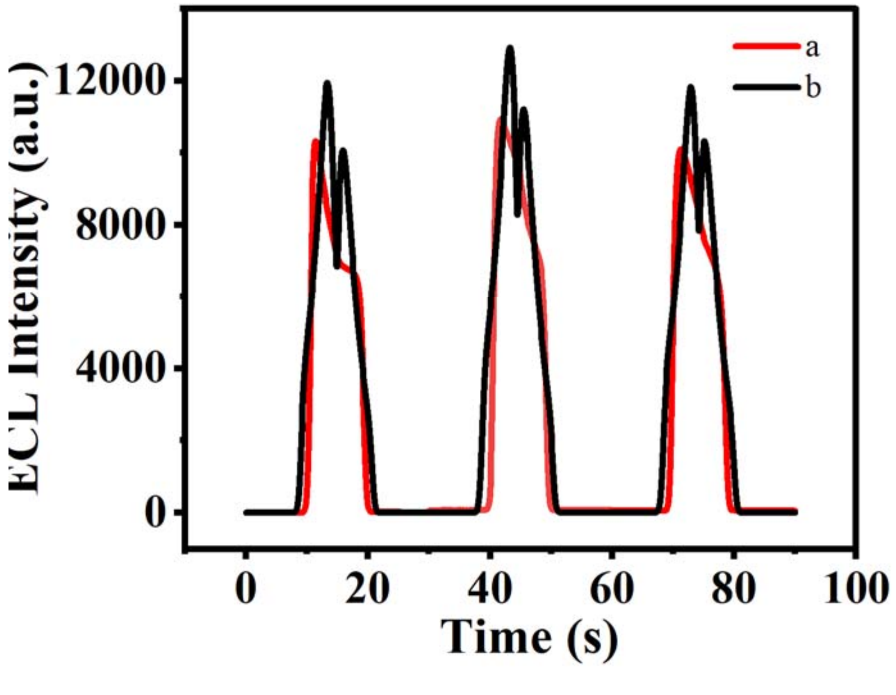

Figure S7. ECL curves of (a) CDs (15.62 mg/mL) and (b) TPrA (1.43 mg/mL) in the solution of 0.1 M PBS (pH = 7.4) containing 50 μM $Ru(bpy)_3^{2+}$. The voltage of PMT was set at 600 V.

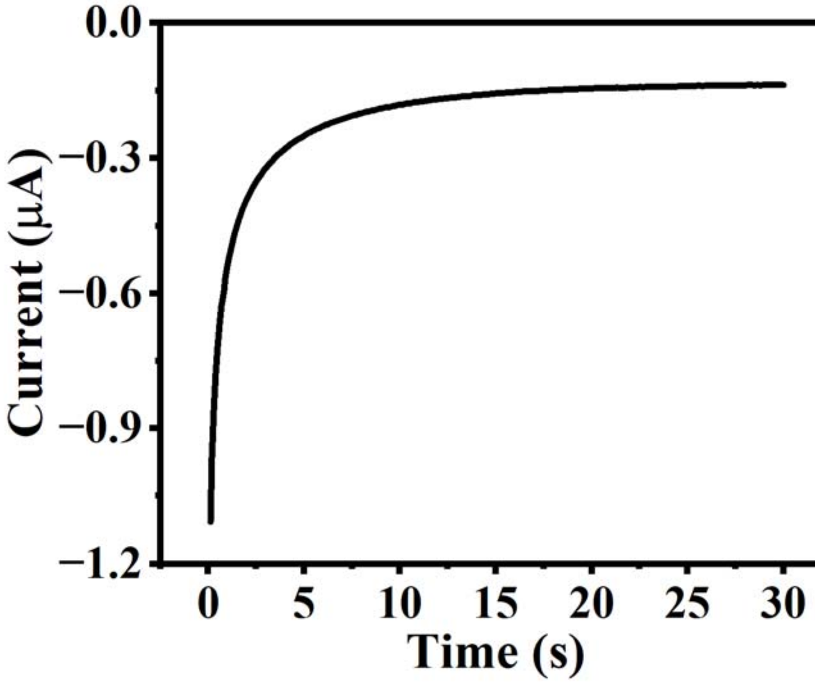

Figure S8. The current-time curve of electrodeposited Au NPs.

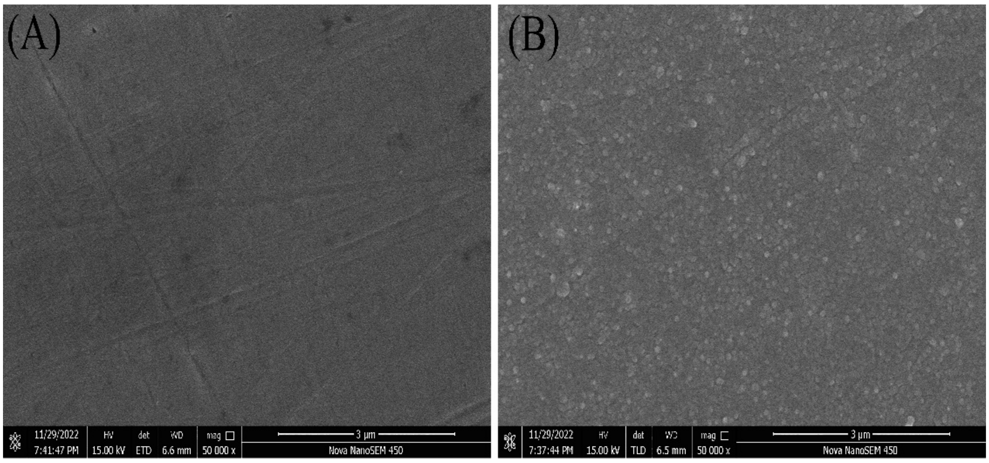

Figure S9. SEM image of electrode surface (A) GCE, (B) Au NPs-GCE.

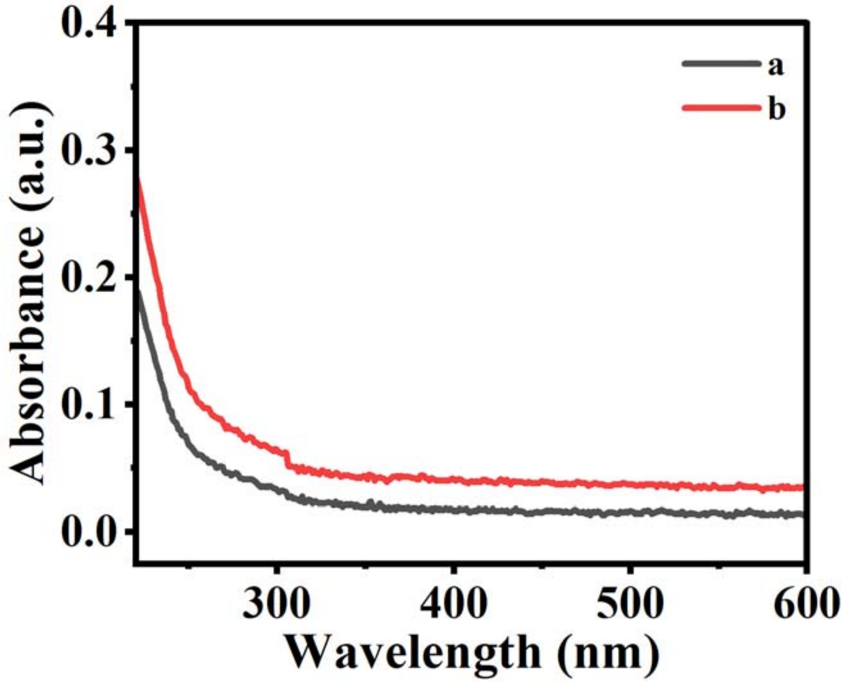

\

Figure S10. UV–vis absorption spectrum of (a) HCG-Ab_1 (0.016 mg/mL) and (b) a solution contains the excess HCG-Ab_1 of the constructed immunosensor.

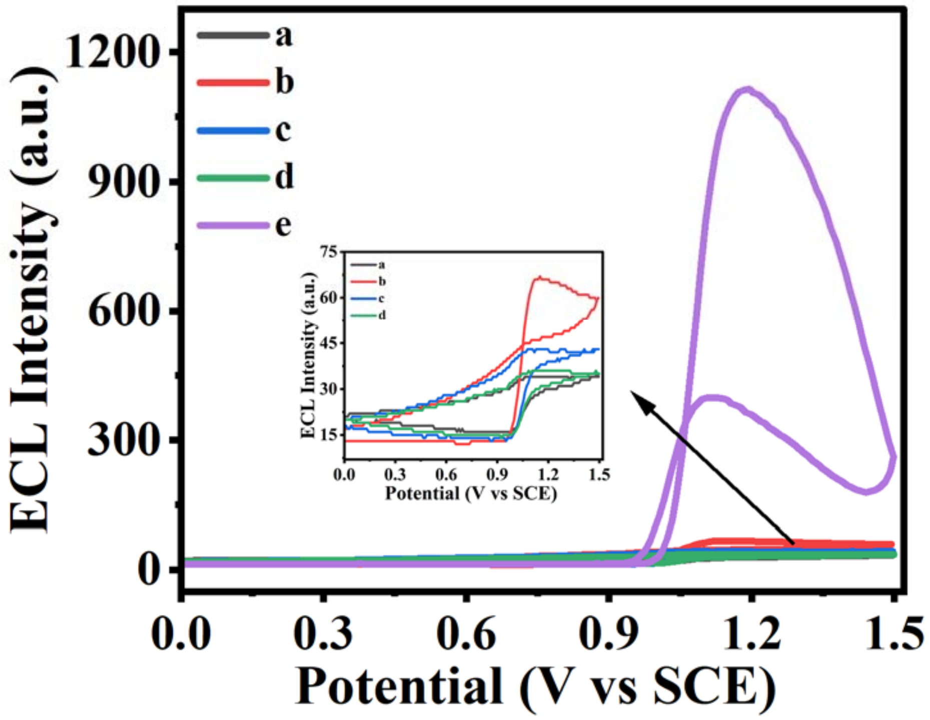

Figure S11. The ECL spectra of electrodes modified with different substances(a) GCE, (b) Ab_1/Au/GCE, (c) BSA/Ab_1/ Au/GCE, (d) Ag/ BSA/Ab_1/ Au/GCE and (e) Ab_2-CDs@dSiO_2 NPs / Ag/ BSA/Ab_1/ Au/GCE in a solution of 0.01 M PBS (pH = 7.4) containing 50 μM $Ru(bpy)_3^{2+}$. The voltage of PMT was set at 800 V.

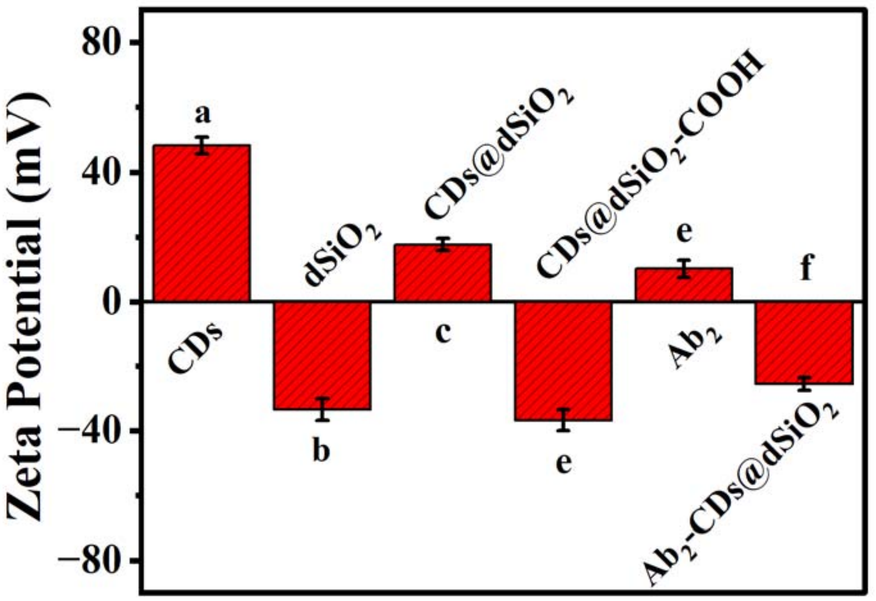

Figure S12. Zeta potential of (a) CDs, (b) $dSiO_2$, (c) CDs@$dSiO_2$, (d) CDs@$dSiO_2$-COOH, (e) Ab_2, and (f) CDs@$dSiO_2$-Ab_2 in the solution of 0.01 M PBS (pH = 7.4).

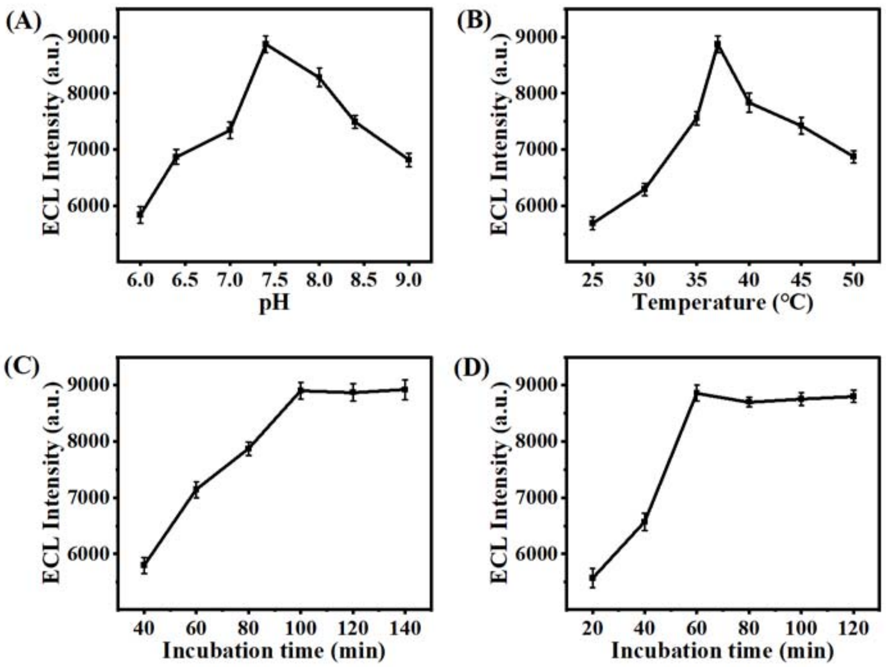

Figure S13. Optimization of (A) pH of PBS. (B) Incubation temperature. (C) Incubation time for Ab_1 bind to the electrode. (D) Specific binding time between the Ag and Ab_1. (Error bars: SD, n = 3). The voltage of PMT was set at 800 V.

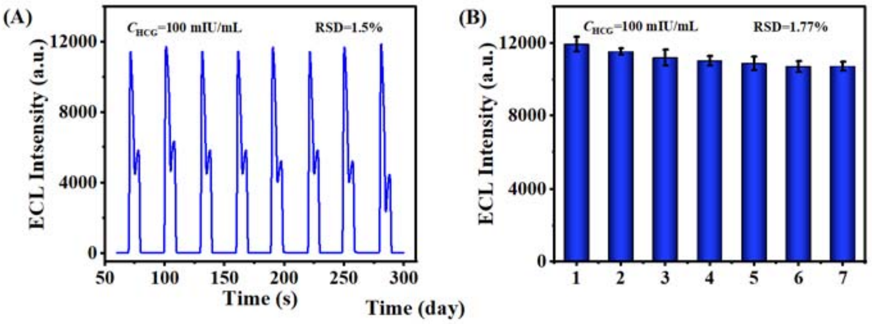

Figure S14. (A) Performance stability and (B) Storage stability of the ECL immunosensor. (Error bars: SD, n = 3). The voltage of PMT was set at 800 V.

Table S1. Comparison of analytical performances with other reported HCG bioassays.

Detection Method	Linear Range	Detection Limit	Ref.
Immunochromatographic strip	0.5-500 mIU/mL	0.22 mIU/mL	[1]
Surface-enhanced Raman scattering	0.5~200 mIU/mL	0.18 mIU/mL	[2]
Chemiluminescence	0.1~10 mIU/mL	0.06 mIU/mL	[3]
Electrochemiluminescence	0.001~500 mIU/mL	0.0003 mIU/mL	[4]
Electrochemiluminescence	0.001~500 mIU/mL	0.00033 mIU/mL	[5]
Electrochemiluminescence	0.0005~500 mIU/mL	0.00019 mIU/mL	Herein

Table S2. Analytical results for HCG detection in human serum specimens.

Sample	Initial Human Serum	Added (mIU/mL)	Measured (mIU/mL)	Average (mIU/mL)	RSD (%)	Recovery (n=3, %)
Serum 1	<LOD	5.00	4.48<br>4.87<br>4.48	5.72	4.4	100.1
Serum 2	<LOD	15.00	13.36<br>13.88<br>15.20	14.15	6.7	99.94
Serum 3	<LOD	25.00	24.4<br>25.87<br>26.02	25.43	2.3	100

References

1. Wang, X.; Xue, C.H.; Yang, D.; Jia, S.T.; Ding, Y.R.; Lei, L.; Gao, K.Y.; Jia, T.T. Modification of a nitrocellulose membrane with nanofibers for sensitivity enhancement in lateral flow test strips. *Rsc Advances.* **2021**, *11*, 26493–26501.https://doi.org/10.1039/d1ra04369b.
2. Wen, G.Q.; Liang, X.J.; Liu, Q.Y.; Liang, A.H.; Jiang, Z.L. A novel nanocatalytic SERS detection of trace human chorionic gonadotropin using labeled-free Vitoria blue 4R as molecular probe. *Biosens. Bioelectron.* **2016**, *85*, 450–456.https://doi.org/10.1016/j.bios.2016.05.024.
3. Lei, J.; Jing, T.; Zhou, T.; Zhou, Y.; Wu, W.; Mei, S.; Zhou, Y. A simple and sensitive immunoassay for the determination of human chorionic gonadotropin by graphene-based chemiluminescence resonance energy transfer. *Biosens. Bioelectron.* **2014**, *54*, 72–77.https://doi.org/10.1016/j.bios.2013.10.033.
4. Zhang, A.; Guo, W.W.; Ke, H.; Zhang, X.; Zhang, H.; Huang, C.S.; Yang, D.P.; Jia, N.Q.; Cui, D.X. Sandwich-format ECL immunosensor based on Au star@BSA-Luminol nanocomposites for determination of human chorionic gonadotropin. *Biosens. Bioelectron.* **2018**, *101*, 219–226.https://doi.org/10.1016/j.bios.2017.10.040.
5. Qin, D.M.; Jiang, X.H.; Mo, G.C.; Zheng, X.F.; Deng, B.Y. Electrochemiluminescence immunoassay of human chorionic gonadotropin using silver carbon quantum dots and functionalized polymer nanospheres. *Microchim. Acta.* **2020**, *187*, 1–13.https://doi.org/10.1007/s00604-020-04450-0.
